# Rapid Covalent Bonding of Walnut Protein Isolates to EGCG: Unveiling the Ultrasound-Assisted Ratio Optimization, Binding Mechanism, and Structural–Functional Transformations

**DOI:** 10.3390/foods14071204

**Published:** 2025-03-29

**Authors:** Yuanyuan Wei, Liping Sun, Ying Gu, Yongliang Zhuang, Gaopeng Zhang, Xuejing Fan, Yangyue Ding

**Affiliations:** 1 Faculty of Food Science and Engineering, Kunming University of Science and Technology, Kunming 650500, China; shikewyy@163.com (Y.W.); lpsun@kmust.edu.cn (L.S.); guying@kust.edu.cn (Y.G.); ylzhuang@kmust.edu.cn (Y.Z.); fanxuejing@kust.edu.cn (X.F.); 2 College of Food Science and Technology, Zhejiang University of Technology, Hangzhou 310014, China; zhanggaopeng@zjut.edu.cn

**Keywords:** walnut protein isolates, polyphenol, ultrasonic cavitation effect, interaction mechanism, covalent conjugate, functionality

## Abstract

The application of walnut protein isolate (WPI) and polyphenols is usually limited by low solubility. To solve the above problem, the impact of the alkaline treatment method and the ultrasound-assisted alkaline treatment method on the structural and functional properties of protein–polyphenol covalent complexes (WPI–(–)-epigallocatechin-3-gallate (EGCG), UWPI–EGCG, respectively) was explored. Fourier transform infrared spectroscopy and fluorescence spectroscopy indicated that the covalent binding of EGCG to WPI altered the secondary and tertiary structures of the protein and increased its random coil content. In addition, the UWPI–EGCG samples had the lowest particle size (153.67 nm), the largest absolute zeta potential value (25.4 mV), and the highest polyphenol binding (53.37 ± 0.33 mg/g protein). Meanwhile, WPI–EGCG covalent complexes also possessed excellent solubility and emulsification properties. These findings provide a promising approach for WPI in applications such as functional foods.

## 1. Introduction

The walnut (*Juglans regia* L.), a member of the *Juglandaceae* family, is a widely distributed deciduous tree known for its globally recognized and nutritious nuts [1]. Various parts of the plant have remarkable functional properties. As the foremost walnut producer, China’s walnut industry focuses primarily on agricultural raw materials and oil extraction. A byproduct of this process, walnut meal, contains important amino acids and 40% to 80% high-quality proteins, including glutelin, globulin, and albumin [2]. Despite its superior nutritional value over other plant and grain proteins, walnut protein (WP) faces solubility issues due to its high glutenin content, which is approximately 70% [3]. The poor solubility adversely affects the protein’s functional characteristics (oil-holding capacity and foaming and emulsification capabilities). Often underutilized and relegated to animal feed or waste disposal, WP remains an inexpensive, protein-rich byproduct [4]. Therefore, there is an urgent need to develop innovative technologies to improve the functionality of WP.

Polyphenols, a diverse class of natural plant compounds that includes phenolic acids, flavonoids, and tannins, are an integral part of the human diet and are expected to interact with proteins in various ways [5]. Protein–polyphenol interactions are governed by non-covalent interactions and the formation of covalent bonds [6]. Covalent bonds between proteins and polyphenols are distinguished by their enhanced durability and stability, which are typically established by non-enzymatic (such as alkali reactions and free-radical transfer) and enzymatic (mediated by polyphenol oxidase and laccase) pathways [7]. Alkaline reactions are a typical non-enzymatic approach for conjugating polyphenols to proteins. Under alkaline conditions, polyphenols are susceptible to oxidation in the presence of oxygen, leading to the formation of semiquinone radicals that are subsequently rearranged into quinones. These quinones, upon oxidation, readily engage with nucleophilic residues, such as Lys or Cys on protein side chains, forming C–N and C–S bonds, respectively [8]. In particular, EGCG, as the most biologically active major component of tea polyphenols, was found in walnut kernel extract at a high level of 563.45 mg GAE/g. EGCG binds to dietary proteins and is likely to play a significant role in modifying WP [9]. The interaction of polyphenols with proteins facilitates structural reorganization, particularly by increasing the flexibility of the molecular structure of proteins, which in turn, improves the techno-functional characteristics (solubility, emulsification, gelling, and foaming capabilities) [10].

However, the structural and functional characteristics of plant proteins are generally more sensitive to pH fluctuations than those of animal proteins, which affects the binding efficiency of plant proteins to polyphenols using alkaline methods [11]. In addition, the alkaline reaction process is long, making it unsuitable for commercial applications. Walnut protein isolates (WPIs) have a compact structure, which may further limit their ability to interact with polyphenols. More critically, traditional pH-shifting techniques also tend to consume excessive energy, and a prolonged exposure of polyphenols to alkaline conditions can result in adverse effects [12]. In particular, under highly alkaline conditions, polyphenols are susceptible to rapid degradation, dimerization, oxidation, and isomerization [13]. Therefore, it is essential to improve the binding efficiency between proteins and polyphenols.

It is well-recognized that the structural features of proteins have a key influence on their ability to bind polyphenols. Among the amino acids, Pro residues significantly increase the capacity of polyphenols to bind to proteins by inducing a conformational shift toward more irregular folding or extension structures [14]. The increased unfolding of the secondary and tertiary structure of a protein correlates with an increased structural flexibility, which in turn, increases the accessibility of binding sites and promotes protein–phenolic interactions [7]. In addition, the creation of covalent bonds between proteins and phenolics is primarily determined by the availability and abundance of nucleophilic protein side chains, like Lys and Cys.

**Figure 1:**
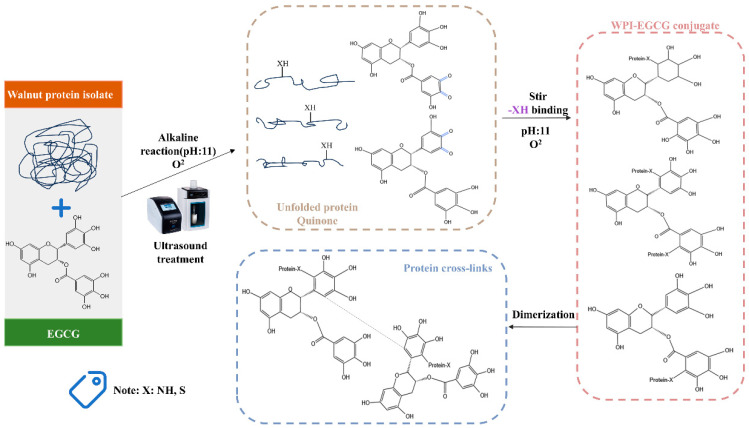
Mechanism of ultrasound-assisted EGCG modification of walnut protein isolate.

Recognized as an energy-efficient and environmentally friendly non-thermal processing technique, ultrasound uses cavitation and mechanical forces to disrupt the hydrogen bonds, electrostatic interactions, and hydration that stabilize protein molecules [15]. This disruption facilitates the denaturation of protein structures, revealing the sequestered hydrophobic amino acid residues, notably Pro. Studies have shown that sonication causes a decrease in the α-helix of WPs and an increase in β-folds, β-sheets, and random coils [16]. The protein’s cryptic hydrophobic regions become more visible due to the reduction in α-helix, potentially increasing the binding affinity between WPs and polyphenols. Figure 1 illustrates the process of EGCG modification of proteins with ultrasonic assistance.

The purpose of this study was to develop WP–EGCG conjugates with enhanced solubility and emulsification using the ultrasound-assisted alkali method. Covalent complex formation and WP structural changes were investigated using multispectral analysis and molecular docking to decode the effects on particle size, solubility, and emulsification properties. The obtained results highlight an effective approach to improve the techno-functional performance of WP, thereby potentially expanding the applications of WP sources in the food industry.

## 2. Materials and Methods

### 2.1. Materials

Walnut (*Juglans regia* L.) meal powder was sourced from the Yunnan Academy of Forestry and Grassland (Kunming, China). EGCG was purchased from Yuanye Bio-Technology Co. (Shanghai, China). All additional chemicals used in the study were acquired from Macklin Biochemical Technology Co. (Shanghai, China) and were of an analytical grade.

### 2.2. Preparation of WPI

Alkaline-extracted WPI was prepared following established protocols [9]. The walnut meal was defatted with *n*-hexane. Defatted walnut meal was combined with distilled water (1:15, *w*/*v*), and the pH was adjusted to 11 using 1.0 mol/L NaOH. The mixture was stirred at an ambient temperature for 2 h, after which the supernatant was separated by centrifugation at 4500 r/min for 30 min. The resulting precipitate was dispersed in deionized water and extracted twice in the same way. The pH was then lowered to 4.5 using 1.0 mol/L HCl, and after settling, the supernatant was centrifuged again at 4500 r/min for 30 min. The sediment was neutralized and lyophilized to obtain WPI.

### 2.3. Preparation of WPI–EGCG Conjugates

WPI–EGCG conjugates were prepared following the method described by Prodpran et al. [17]. Briefly, 1 g WPI was mixed in 100 mL deionized water at pH 11 with stirring for 1 h to ensure full dissolution. Subsequently, 0.5 g EGCG was mixed in 50 mL of deionized water (10 mg/mL) with the pH adjusted to 9.0 using NaOH. EGCG was added to the WPI solution in different mass ratios, and the pH was adjusted to 11. Two equal portions of the prepared WPI–EGCG mixture were separated. One was immediately exposed to air and stirred for 2 h (WPI–EGCG: 1:50, 1:25, 1:12.5, *w*/*w*), and the other was exposed to 450 W ultrasound for 15 min, followed by reaction with oxygen for 2 h (UWPI–EGCG: 1:50 U, 1:25 U, 1:12.5 U). The WPI–EGCG conjugates were then dialyzed in a dialysis bag (3500 Da) for 36 h with water changes every 4 h, after which the dialysate was lyophilized for a subsequent analysis of the structural and functional properties.

### 2.4. Determination of Grafted Polyphenol Contents

One milliliter of the WPI–EGCG conjugates solution (1 mg/mL) was combined with 5 mL of a 10% Folin–Ciocalteu reagent and allowed to react for 4 min. Then, 4 mL of Na_2_CO_3_ solution were added, and the reaction was continued for 2 h, avoiding light. Absorbance at 760 nm was determined at the end of the reaction. The total polyphenol content was calculated based on the EGCG calibration curve (*y* = 7*x* + 0.05, R^2^ = 0.991).

### 2.5. Free Sulfhydryl Content

Elman’s reagent (DTNB) was used to determine the free sulfhydryl groups in the proteins [18]. In short, 1 mL of a protein sample (5 mg/mL) was combined with 5 mL of Tris–glycine buffer (pH 8.0; 0.086 mol/L Tris, 0.09 mol/L glycine, and 0.004 mol/L EDTA). To initiate the reaction, 0.04 mL of DTNB reagent (0.02 g DTNB in 5 mL of Tris–glycine buffer) were added to the sample solution, mixed quickly, and left for 20 min in the dark. Finally, the absorbance was recorded at 412 nm.

The formula for calculating the free sulfhydryl content of a sample is as follows:

(1) $$\delta (\mu \mathrm{mol}/g)=\frac{10^{6}{\times A}_{412}\times D}{1.36\times 10^{4}\times C}$$

where *δ* represents the free sulfhydryl content (μmol/g protein), 1.36 × 10^4^ is the molar extinction coefficient, *C* denotes the protein concentration of the sample (mg/mL), and *D* is the dilution factor.

### 2.6. Free Amino Content

The content of free aminogroup in samples was assessed using *o*-phthalaldehyde (OPA) [19]. To prepare the OPA reagent, 80 mg of OPA was dissolved in 2 mL of methanol, 50 mL of sodium borate buffer (0.1 mol/L), 5 mL of sodium dodecyl sulfate (SDS, 20%), and 0.2 mL of β-mercaptoethanol, followed by volume setting into a 100 mL volumetric flask. The WPI–EGCG conjugate solution (0.4 mL) was combined with the OPA reagent (8 mL) and reacted at 35 °C for 3 min. The absorbance was recorded at 340 nm. L-Leu served as the amino acid standard, and the sample concentration was determined based on the standard curve obtained (*y* = 0.0001*x* + 0.0396, R^2^ = 0.9933).

### 2.7. SDS-PAGE

SDS-PAGE analysis was performed as previously reported [20], with minor modifications. The sampling buffer (5×) was mixed with a protein sample solution (5 mg/mL) at 1:1 (*v*/*v*) and later boiled in boiling water for 8 min. Next, 15 μL of the test protein solution and 3 μL of the marker (protein ladder) were loaded into the wells for electrophoresis. The procedure began at a voltage of 80 V, which was increased to 120 V as the samples entered the separation gel. After electrophoresis, the gel was stained with Coomassie R-250 for 2 h, followed by decolorization, and images of the gel were captured digitally.

### 2.8. Ultraviolet–Visible (UV–Vis) Spectra Analysis

The solution was diluted (0.2 mg/mL) with PBS (0.01 mol/L, pH 7.0). Experiments were performed using a UV spectrophotometer (UV-1700, Shimadzu Co., Ltd., Tokyo, Japan). The UV absorption spectra of the WPI–EGCG conjugates were measured in the wavelength range of 230–360 nm and moderate velocities, and the data collection interval was set at 0.5 nm.

### 2.9. Fluorescence Spectra Analysis

The fluorescence spectra were estimated following the approach outlined by Wang et al. [21] with slight modifications. After diluting the WPI–EGCG conjugates to 0.2 mg/mL in PBS (10 mmol/L, pH 7.4), the intrinsic fluorescence spectra of the WPI–EGCG conjugates were determined using a fluorescence spectrophotometer (F-4600, Hitachi, Tokyo, Japan). The excitation wavelength was set at 295 nm, and the emission wavelength range spanned from 300 to 400 nm. The slit width was maintained at 5 nm, with a scan rate of 1200 nm/min and a scan voltage of 700 mV. Three scans were conducted repeatedly for each sample.

### 2.10. Fourier Transform Infrared Spectroscopy (FTIR) Analysis

The FTIR spectra of the WPI–EGCG conjugates were determined using a spectrometer (Thermo Fisher Scientific Inc., Waltham, MA, USA). The FTIR spectra of the WPI–EGCG conjugates ranged from 4000 to 400 cm^−1^ (64 scans).

### 2.11. Determination of Particle Size and Zeta Potential

Ten mg of the WPI–EGCG conjugates were dissolved in 10 mL of distilled water. The particle size and zeta potential of the WP–EGCG conjugates were analyzed using the dynamic light-scattering method with a Zetasizer Nano ZS90 (Malvern Instruments, Malvern, UK).

### 2.12. Scanning Electron Microscopy (SEM) Analysis

The WP–EGCG conjugates were sprinkled onto a double-sided conductive adhesive fixed on the sample disk, followed by spraying a thin layer of platinum at 20 mA. The morphology of the protein was observed using a scanning electron microscope (Thermo Scientific Apreo 2C; Thermo Scientific, Waltham, MA, USA) at an accelerating voltage of 5 kV, with sample magnifications of 1000×.

### 2.13. Atomic Force Microscopy (AFM) Analysis

The microstructures of the WPI and WPI–EGCG conjugates were observed using AFM (Bruker Dimension Icon; Bruker, Billerica, MA, USA) according to the method of Chang et al. [22] with slight modifications. An appropriate amount of the sample was dropped on a clean mica sheet and dried overnight at room temperature, after which the sample was observed in the PeakForce Tapping mode.

### 2.14. Protein Solubility

Protein solubility was assessed following a protocol adapted from a study by Wang et al. (2016) [20] with minor adjustments. Five mg of WPI–EGCG conjugates were weighed and dissolved in 5 mL of deionized water. After thorough mixing, the protein solutions were centrifuged at 9000 r/min for 15 min under ambient conditions. The protein concentrations in both the pre-centrifugation samples and the post-centrifugation supernatants were quantified using the Coomassie brilliant blue method, with bovine serum albumin (BSA) as the reference standard. Protein solubility was calculated as the ratio of protein in the supernatant to the total protein concentration before centrifugation, expressed as a percentage.

### 2.15. Determination of Emulsifying Properties

The sample powder was weighed to prepare 15 mL of protein solution with a mass fraction of 1% (*w*/*v*), and 5 mL of triglyceride were added. The emulsion was prepared by high-speed homogenization with rapid mixing at 10,000 r/min for 1 min and then circulated three times. Immediately, 0.1 mL of the emulsion was added into 10 mL of 0.1% (*w*/*v*) SDS and mixed well with vibration. Immediately, the absorbance at 500 nm was recorded as *A*_0_ using a UV spectrophotometer. After reslting 30 min, 0.1 mL of the emulsion was transferred into 10 mL of 0.1% SDS and mixed well with vibration. The absorbance was recorded as *A*_30_. The emulsifying activity index (EAI) and emulsion stability index (ESI) were calculated as follows:

(2) $$\mathrm{EAI}(m^{2}/g)=\frac{2\times 2.303\times A_{0}\times \mathrm{DF}}{C\times \theta \times \varphi \times 10^{4}}$$

(3) $$\mathrm{ESI}(\%)=\frac{A_{30}}{A_{0}}$$

where *DF* represents the dilution factor, *C* is the protein concentration (g/mL), *θ* denotes the oil phase volume fraction (20%), *φ* is the optical path (1 cm), *A*_0_ is the absorbance measured at the initial time, and *A*_30_ is the absorbance at 30 min.

### 2.16. Molecular Docking

To identify potential binding sites between WPI and EGCG and to uncover the molecular interaction mechanisms, the representative crystal structure of WPI (glutelin NCBI Protein ID: XP_018828122.2, 11S globulin PDB code: Q2TPW5, and 2S albumin PDB code: P93198) [23] and the EGCG (PubChem CID: 65064) structures were obtained from the RCSB Protein Data Bank (https://www.rcsb.org/ accessed on 17 December 2024) and PubChem database (https://pubchem.ncbi.nlm.nih.gov/ accessed on 17 December 2024). Using AutoDock 4.2, the docking sites between glutelin, 11S globulin, 2S albumin, and EGCG were identified based on the lowest binding energy configurations. The resulting interactions were then visualized using PyMol 2.4 software for further analysis.

### 2.17. Statistical Analysis

All the experiments were replicated at least three times. Data are presented as the mean ± standard deviation. Statistical analysis was performed using a one-way analysis of variance (ANOVA) and Duncan’s test for mean separation. Origin 2021 software (Origin Lab Co., Northampton, MA, USA) was used for all statistical analysis. *p* < 0.05 was regarded as statistically significant.

## 3. Results and Discussion

### 3.1. Polyphenol Contents

**Figure 2:**
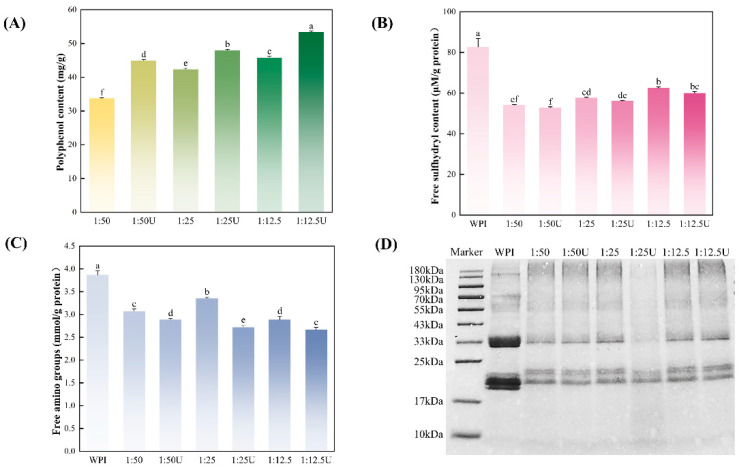
(**A**) Polyphenol binding equivalents of walnut protein isolate–EGCG conjugates (WPI–EGCG) under different treatments, (**B**) free sulfhydryl content, (**C**) free amino content, and (**D**) SDS-PAGE profiles. Means with different letters show significant differences (*p* < 0.05).

The polyphenol contents in the WPI–EGCG conjugates prepared using alkali and ultrasound-assisted alkali treatment methods are shown in Figure 2A. Statistical analysis revealed that the binding of polyphenols increased from 33.74 ± 0.20 mg/g to 45.82 ± 0.38 mg/g (1:12.5 group) as the polyphenol concentration increased (*p* < 0.05). It should be noted that the polyphenol binding capacity of the UWPI–EGCG conjugates was stronger when compared with the WPI–EGCG conjugates, reaching a maximum of 53.37 ± 0.33 mg/g, which may be due to the turbulent forces and free radicals generated by ultrasound. Turbulent forces effectively disrupted the protein structure, unveiling the binding sites (sulfhydryl and amino groups) and thereby promoting EGCG grafting [24], while free radicals were able to catalyze the oxidation of polyphenols into quinones under alkaline conditions (pH ≥ 9.0), which later react with WPI side-chain amino acids. These results are consistent with the study by Iscimen et al. [25], who reported that the sonication amplitude positively correlated with the polyphenol grafting rate (*p* < 0.05). Specifically, the polyphenol binding equivalents of the fava bean protein–grape leaf polyphenol conjugates increased from 57.3 ± 0.3% to 72.2 ± 0.3% as the sonication amplitude increased, further validating the critical role of ultrasonic energy input in enhancing conjugation efficiency.

### 3.2. Free Sulfhydryl and Amino Contents Analysis

The reactive chemical nature of free sulfhydryl and amino groups in proteins is crucial for determining the redox state and functional characteristics. By monitoring changes in the contents of these groups, the extent of conjugation between proteins and polyphenols can be evaluated. As shown in Figure 2B,C, significant declines in the sulfhydryl and amino groups were observed across all WPI–EGCG conjugates compared to the WPI group (*p* < 0.05), where the sulfhydryl and amino groups were reduced by 30.69 μM/g and 1.20 mmol/g, respectively. In addition, the free sulfhydryl content in the covalent complexes increased as the mass ratio of EGCG to WPI increased, which may be attributed to the difference in the EGCG content in the conjugates and the spatial site-blocking generated by the complex chemical structure of EGCG. The covalent binding to the protein limited the modification of the protein by the cavitation effect [26]. However, the free sulfhydryl groups in the sonicated groups showed a slight reduction compared to those in the non-sonicated groups (*p* > 0.05). This indicates that sonication loosens the protein structure, causing previously buried sulfhydryl groups to become more accessible on the surface, and the EGCG could interact with these exposed sulfhydryl groups. A similar trend was observed for the free amino groups, which suggests that EGCG can bond to sulfhydryl and amino groups within the protein structure via C–N or C–S bonds [27]. In the present study, the free amino groups decreased to a minimum value of 2.67 ± 0.05 mmol/g (1:25 U group), which suggests that sonication enhances the cross-linking between the EGCG and free amino groups. These findings align with the study of Sun et al. [28], who showed that the sonochemical action of sonication leads to an increased generation of hydroxyl radicals. Increased oxidation of free amino groups by hydroxyl radicals results in a higher participation of these groups in reactions with polyphenols through C–N bonds [29].

### 3.3. SDS-PAGE Analysis

The effect of EGCG on WPI was further analyzed using reduced SDS-PAGE (Figure 2D). WPI (lane 1) had four regional bands, mainly at 130, 55–70, 30–37, and 18–23 kDa, respectively. The molecular weight of the WPI–EGCG conjugates was significantly elevated compared to WPI, with protein bands migrating upward to 18 and 30 kDa and becoming lighter and finer, suggesting that covalent binding to EGCG increased the molecular weight of the WPI. This finding was consistent with a previous study on the conjugates of bovine bone proteins with various polyphenolic compounds [30]. In addition, there is no significant difference among the covalent complexes or between the sonicated and non-sonicated treatments. This indicates that sonication does not create new small molecular subunits. Therefore, water-bath sonication cannot change the primary structure of proteins. The same conclusion was reached by O’Sullivan et al. [31]. This phenomenon may be because proteins with strong intermolecular interactions limit the unfolding of the spatial structure by ultrasonic cavitation effects and shear forces, thus greatly protecting their primary structure from disruption [32].

### 3.4. UV–Vis Spectra

**Figure 3:**
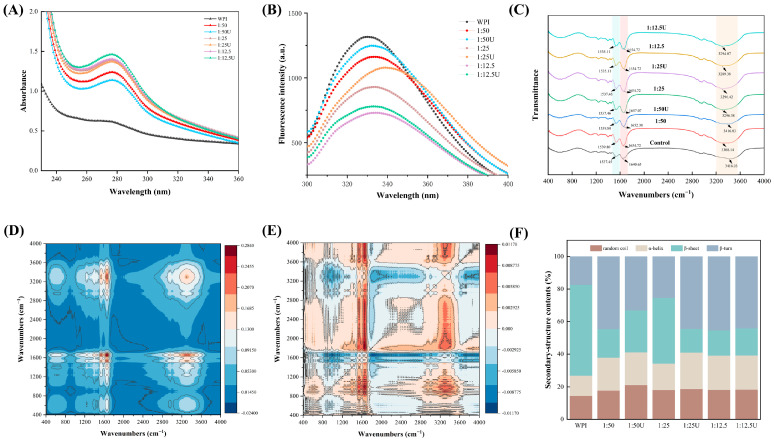
Protein structural changes in WPI and WPI–EGCG conjugates. (**A**) UV–vis analysis, (**B**) the intrinsic fluorescence spectra, (**C**) FT-IR spectrum, (**D**) synchronous 2DCOS-FTIR spectra, (**E**) asynchronous 2DCOS-FTIR spectra, (**F**) proportion of secondary structure.

The interactions between proteins and biomolecular ligands, as well as the tertiary structural alterations in proteins, can be elucidated using UV–vis absorption spectroscopy. Figure 3A displays the UV–vis absorption spectra of WPI both before and after its interaction with EGCG. Upon complexation with EGCG, a notable enhancement in the UV-absorption intensity of WPI was observed, with the maximum absorption peak shifting to approximately 280 nm. This increase in UV-absorbed intensity was likely due to the EGCG-induced extension of the WPI peptide chains, exposing aromatic residues like Tyr and Trp [33]. The peak observed at about 280 nm corresponds to the π–π* electronic transition of these aromatic amino acids, including Phe [34]. The intensified UV absorption peaks in the sonicated groups suggest that WPI undergoes a more pronounced modification during ultrasound-assisted alkaline treatment when compared with the alkaline treatment alone, which may lead to further perturbations in the protein’s tertiary structure [35]. Collectively, these findings imply that covalent binding to EGCG potentially induces the unfolding and structural reorganization of WPI.

### 3.5. Fluorescence Spectroscopy Analysis

Fluorescence spectroscopy is an effective method for observing shifts in the microenvironmental polarity of aromatic amino acids such as Tyr and Trp, monitoring the alteration of the tertiary structure of protein. Figure 3B shows that WPI exhibited strong fluorescence emission, and the fluorescence changes after the addition of EGCG, which were manifested by the decrease in its fluorescence intensity with the increase in the addition of EGCG and the redshift of the maximum absorption wavelength (redshifted from 330.02 nm to 338.71 nm in the 1:25 U group). It suggested that the microenvironmental polarity surrounding Trp underwent a shift when its residues, originally nestled in the hydrophobic core, became exposed to the hydrophilic outer environment, and the hydrophobic groups, such as Trp, Tyr, and Phe residues within WPI, played a role in the covalent binding [36]. In addition, our study found that the fluorescence intensity of 1:50 U, 1:25 U, and 1:12.5 U was greater than that of 1:50, 1:25, and 1:12.5, respectively, which may be associated with the exposure of Trp/Tyr residues in the unfolded protein due to sonication. This result was in agreement with Tong et al. [37]. However, a different conclusion was reached by Zheng et al. [38], who found that sonication of protein–polyphenol couplings further decreased the fluorescence intensity. These inconsistencies across studies may be due to differences in sample type and experimental methodology. At the same time, the maximum redshift occurred in the 1:25 U group, suggesting that ultrasound promotes interaction between the WPI and EGCG.

### 3.6. FT-IR Analysis

FTIR is a spectroscopic technique for analyzing alterations in the chemical bonds formed between protein and polyphenol conjugates (Figure 3C–E). In WPI, the peak locations of amide II and amide I bands were 1640.65 and 1537.45 cm^−1^, respectively. In WPI–EGCG (1:50, 1:25, and 1:12.5), amide II migrated to 1654.72, 1657.07, and 1654.72 cm^−1^, and amide I bands migrated to 1539.80, 1537.46, and 1535.11 cm^−1^, respectively. In UWPI–EGCG (1:50 U, 1:25 U, and 1:12.5 U), amide II bands migrated to 1652.38, 1654.72, and 1654.72 cm^−1^, and amide I bands migrated to 1539.80, 1537.46, and 1535.11 cm^−1^, respectively. The addition of EGCG induced a pronounced redshift in the amide I band. Both WPI–EGCG and UWPI–EGCG conjugates exhibited significant alterations in peak intensities and positions compared to WPI. The findings showed that conjugating WPI with EGCG alters the secondary structure of WPI. Additionally, the combined effects of polyphenol grafting and sonication lead to significant rearrangements and alterations in the protein structure. At the same time, the findings suggest that EGCG engages with the C=O and C–N functional groups within WPI, leading to the formation of conjugates. Furthermore, compared with WPI, the conjugates displayed a notable blueshift at 3416.03 cm^−1^. This result was consistent with the study of He et al. [39]. It indicated that the hydroxyl content of proteins increases upon binding to polyphenols via covalent bonding, thus showing enhanced hydroxyl feature absorption enhancement, further demonstrating that EGCG covalently binds to WPI.

The secondary structure of the WPI–EGCG conjugates is presented in Figure 3F. The covalent bonding of EGCG primarily caused alterations in the secondary structure of WPI. WPI contained 12.04 ± 0.35% α-helix, 57.05 ± 1.20% β-sheet, 19.54 ± 1.92% β-turn, and 11.37 ± 2.74% random coil content. Adding EGCG increased the content of α-helix (22.94 ± 0.86%), β-turn (45.58 ± 0.13%), and random coil (19.92 ± 1.53%), then decreased the β-sheet content (15.58 ± 2.02%) in WPI. It was shown that, upon the interaction of EGCG with WPI, the structure of the proteins became disordered, and sonication resulted in the creation of more unfolded structures in the conjugates. Wu et al. [40] reached the same conclusion. They indicated that the α-helix and random coil conformations exhibit greater flexibility among the secondary structure, facilitating the protein adsorption and unfolding at the oil–water interface.

### 3.7. Morphological Characters

**Figure 4:**
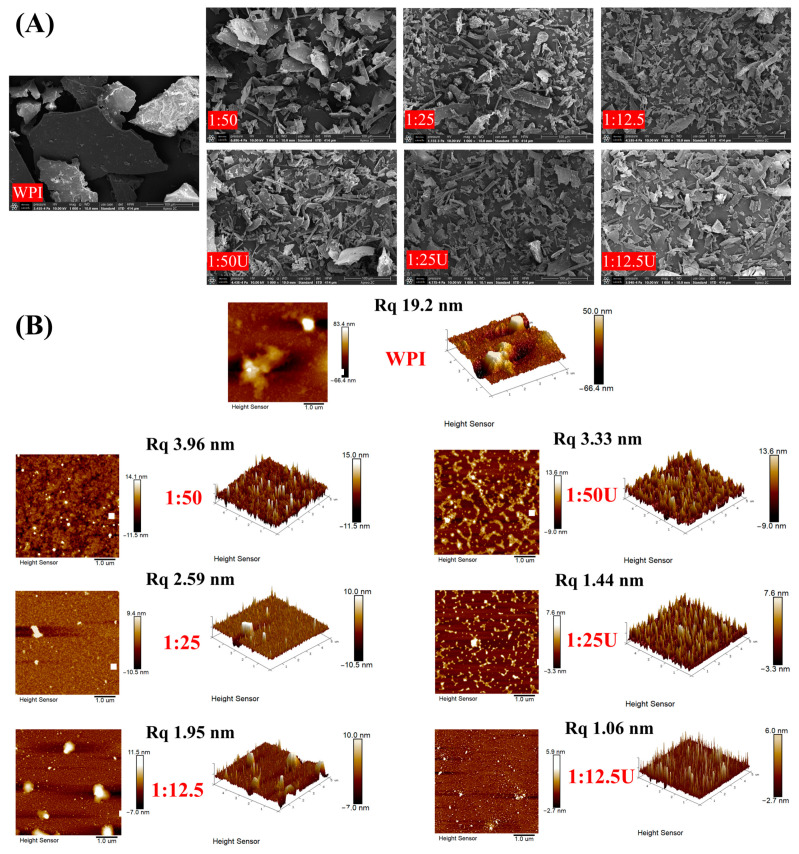
(**A**) SEM images and (**B**) AFM images of walnut protein isolate (WPI) and WPI–EGCG conjugates.

The morphology of WPI and WPI–EGCG conjugates was observed using SEM. As illustrated in Figure 4A, the protein molecules of WPI exhibited a large lamellar structure because they are bound at a high density, and this structure affected their dispersion and solubility. On the contrary, the WPI–EGCG covalent complexes showed irregular shapes of different sizes due to the introduction of EGCG, which destroys the tertiary and quaternary structures of WPI. Furthermore, the new arrangement and aggregation between the individual subunits leads to the disappearance of large pieces of structure [41]. This result aligns with the redshift phenomenon of the preceding fluorescence spectra (Figure 3B), both of which demonstrate the structural unfolding of the complex. In addition, the structure of the covalent complex after sonication was more fragmented, and some fine rod-like structures appeared, which were attributed to the cavitation force and microfluidization exerted by the ultrasound probe disrupting the original structure of the protein, leading to a looser structure of the complex [42].

### 3.8. Surface Topography of WPI–EGCG Conjugates

To show that EGCG has some influence on the structure of WPI, AFM was used to further examine the microscopic morphology of WPI and WPI–EGCG conjugates. As presented in Figure 4B, the radius and height of the WPI–EGCG and UWPI–EGCG conjugates decreased relative to native WPI. The surface roughness (Rq) of native WPI was 19.2 nm, and the addition of EGCG provided strong hydrogen bonding, which turns the large aggregates into more dispersed ones, with the minimum Rq of the complexes being 1.95 nm [43]. The WPI–EGCG conjugates obtained using the ultrasound-assisted method had a minimum Rq of 1.06 nm, with a more dispersed and homogeneous distribution of the structures. This aligned with the findings of Li et al. [44]. This is because ultrasonication transformed the proteins, originally in a disordered aggregation state, into more regular and dispersed aggregates. These results show that EGCG can change the structure of WPI, and these effects are more pronounced when combined with ultrasound-assisted alkaline treatment.

### 3.9. Analysis of Particle Size and Zeta Potential

**Figure 5:**
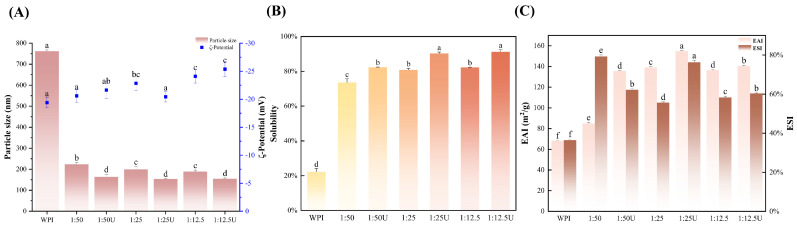
Changes in (**A**) the potential size and zeta potential, (**B**) solubility, and (**C**) emulsification properties. EAI is the emulsifying activity index, and ESI is the emulsion stability index. Means with different letters show significant differences (*p* < 0.05).

As shown in Figure 5A, the particle size of WPI was 761.77 nm, and the particle size of the WPI–EGCG conjugates reduced significantly with a higher EGCG addition; sonication further promoted this phenomenon. The smallest particle sizes were found for 1:25 U and 1:12.5 U, which were 153.67 and 154.77 nm, respectively, which could be attributed to the decrease in the isoelectric point of WPI after covalent binding to EGCG, and the disruption of the protein structure by the cavitation and mechanical forces generated by ultrasound, which reduced the aggregation of WPI and improved the stability of the covalent complexes [45]. A similar finding was reported in the study by Geng et al. [46]. In addition, the reduction in the particle size of the complexes might also be associated with a rise in the absolute zeta potential value (Figure 5A), which provides electrical repulsion, leading to smaller particle sizes [47].

Upon the WPI–EGCG conjugates being generated by alkaline treatment alone, the absolute value of the zeta potential of WPI rose to 24.1 mV. Conversely, when ultrasound-assisted alkaline treatment in combination with EGCG was implemented, the zeta potential of the WPI rose to 25.4 mV. These findings suggest that the WPI–EGCG conjugation not only augments the surface density of phenolic hydroxyl groups on WPI but also amplifies the electrostatic repulsion, thereby enhancing the stability of the covalent complexes [34]. Furthermore, the ultrasound-assisted conjugation with EGCG likely induces protein unfolding and the fragmentation of WPI into smaller particles [42]. This process increases the surface area and exposes more charged molecules, possibly contributing to the observed stability enhancement.

### 3.10. Solubility Analysis

Water solubility is a critical functional characteristic of proteins that affects other functions, including emulsification properties, foaming ability, and gel properties. As shown in Figure 5B, the solubilities of 1:50, 1:25, and 1:12.5 were all higher than the WPI, indicating that WPI conjugation with EGCG improved the solubility of the protein by altering the charge of the protein in the presence of phenolic hydroxyl groups [48]. With increasing amounts of polyphenols, the solubility increased from 22.22 ± 1.9% to 82.23 ± 0.2%. Compared to other polyphenol–protein systems, the solubility enhancement by WPI–EGCG conjugation was more pronounced than walnut protein modified with ellagic acid (+52% solubility at 0.5 mg/mL) [6], but less than pumpkin seed protein–EGCG conjugation (+66% solubility) [49]. This difference may be caused by structural differences. In addition, the solubilities of the sonicated groups were all significantly higher than for the non-sonicated groups, and the maximum solubility was as high as 91.22 ± 1.3%. These results might be because the conjugation reaction of EGCG with WPI increased the surface charge, and its repulsive force was sufficient to inhibit the copolymerization process [6]. Moreover, the binding of EGCG could cause a partial unfolding of the internal architecture of WPI molecules, exposing the internal hydrophilic groups. The cavitation effect produced by the ultrasound-assisted method affected the secondary structure of the protein, breaking the internal bonds among the protein molecules and exposing additional internal hydrophilic groups. This process ultimately enhances the solubility of the protein [50].

### 3.11. Emulsifying Properties

Because of their amphiphilicity and film-forming ability, proteins are often used as emulsifiers in food processing to maintain food quality [51]. Emulsifiability is important for the application of proteins as surfactants. Figure 5C shows the EAI and ESI values for WPI, WPI–EGCG conjugates, and UWPI–EGCG conjugates. Native WPI had a low EAI and ESI of 68.17 ± 0.32 m^2^/g and 36.49%, respectively. Significant improvements were observed in both EAI and ESI after covalent conjugation with EGCG (*p* < 0.05), which may be attributed to the covalent binding of WPI and EGCG providing more carboxyl groups. As the concentration of polyphenols increased, there was an observable pattern of rising and then falling values, with the 1:25 U group exhibiting the maximum value of 154.82 ± 0.56 m^2^/g, representing an 86% increase compared to native WPI. This result was consistent with the results of the fluorescence spectra. Similar conclusions were reached by Ke and Li [52] and Wu, Lin, Zhang, Wang, and Ding [40], who utilized EGCG for covalent binding to soy isolate and soy protein hydrolysate and found that the EAI was positively correlated with the addition of EGCG. However, in contrast to the conclusion that the ESI of WPI–EGCG and UWPI–EGCG was significantly increased (*p* < 0.05) in this experiment, the ESI of soy protein isolate–EGCG decreased relative to that of natural soy protein isolate. Meanwhile, the ESI of soy protein hydrolysate–EGCG, although higher than that of the unmodified soy protein hydrolysate, decreased with the degree of hydrolysis of the soy protein hydrolysate. A decrease in ESI might be due to the lower molecular weight and surface activity, which cannot form a stable interface with the oil. Then, an increase of ESI might be because the polyphenols form a multilayered adsorbent film on the surfaces of the proteins, which improves the capacity of the proteins to lower the interfacial tension between the oil and water and, thus, improves the emulsification stability of the proteins [32]. Similarly, previous studies reported that, when whey protein isolates form covalent bonds with EGCG, the aromatic amino acid residues become more exposed [53]. This heightened exposure enhances the protein’s ability to interact with the oil–water interface, resulting in a higher EAI and ESI of the conjugates than the whey protein [53].

### 3.12. Molecular Docking Analysis

**Figure 6:**
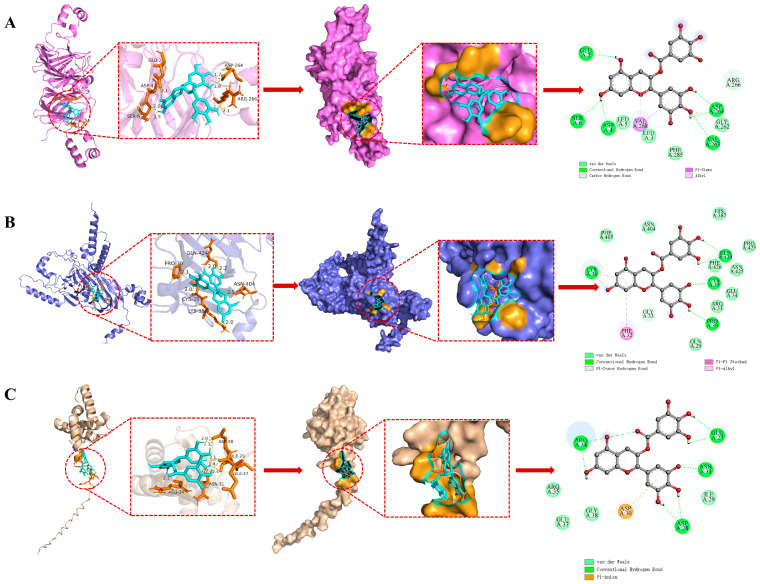
Schematic diagram of the binding process between (**A**) glutelin, (**B**) globulin, and (**C**) albumin proteins and EGCG, including (**left**) molecular docking results of EGCG and proteins, (**middle**) active docking sites, and (**right**) two-dimensional (2D) diagram of the interaction between EGCG and amino acid residues of proteins.

Molecular docking was performed to predict the interactions and potential binding sites between WPI and EGCG. Consequently, we conducted a docking analysis of the three predominant proteins in WPI (glutelin, 11S globulin, and 2S albumin) with EGCG. As presented in Figure 6 and detailed in Table 1, the most stable docking configurations, characterized by the lowest binding energies, were selected for the complexes of glutelin, 11S globulin, and 2S albumin with EGCG. The ranking of binding affinity, from strongest to weakest, was 2S albumin–EGCG with a binding energy of –5.03 kcal/mol, then glutelin–EGCG at –3.66 kcal/mol, and 11S globulin–EGCG at –3.26 kcal/mol. Docking outcomes with binding energies below the threshold of –1.2 kcal/mol are typically deemed plausible, indicating a robust binding interaction between EGCG and WPI.

As shown in Figure 6 (right), for the docking case of glutelin with EGCG, 11 amino acid residues, namely Glu 2, Ser 6, Asp 4, Leu 5, Val 268, Leu 3, Phe 285, Val 263, Gly 262, Asp 264, and Arg 266, were involved in binding interactions, including conventional hydrogen bond, C–H bond, π–sigma, and van der Waals forces. For the docking case of 11S globulin with EGCG, 15 amino acid residues, namely Lys 36, Phe 32, Gly 33, Gln 29, Pro 30, Cys 35, Arg 31, Phe 426, Glu 34, Asn 425, Pro 423, His 387, Asn 404, Phe 405, and Gln 424, were involved in binding interactions involving conventional hydrogen bonding, π–π stacked, π–sigma, π–π hydrogen bond, and van der Waals forces. For the 2S albumin and EGCG, nine amino acid residues, namely Arg 34, Asp 30, Asp 28, Asn 31, Arg 35, Glu 37, Gly 38, Ile 29, and Glu 27, were involved in the binding interactions, including conventional hydrogen bond, π–alkyl, π–anion, and van der Waals forces. The amino acid residues created a hydrophobic pocket that encapsulates the EGCG molecule (Figure 6, middle). Additionally, the findings suggested that EGCG primarily binds to WPI via hydrogen bonds and hydrophobic interactions. These forces are important for stabilizing the position of the EGCG ligand within the active binding sites of glutelin, 11S globulin, and the 2S albumin active binding cavity. This stabilization is crucial for the formation of WPI–EGCG conjugates. Under alkaline conditions, EGCG can be oxidized to a semiquinone radical. This radical is susceptible to nucleophilic substitution by the nitrogen atom, specifically the primary amine group, present in the side chain of certain amino acids [54]. Upon completion of this reaction step, the ligand is capable of reverting to its reduced phenolic form, facilitating hydrogen bond formation with adjacent amino acid residues in the complex. Additional noncovalent interactions at the optimal binding site provide additional stability for EGCG.

## 4. Conclusions

In this study, WPI was covalently bound to EGCG using an alkali treatment and an ultrasound-assisted alkali treatment, and spectroscopy and molecular docking were performed to analyze the interactions between them, as well as to assess the impact of varying mass ratios of EGCG on the covalent complexes. UV–vis and fluorescence spectroscopy revealed that ultrasound assisted in promoting the accessibility of binding sites in WPI. This improvement promoted the rapid formation of C–N and C–S bonds between the WPI (sulfhydryl and amino groups) and EGCG, while also revealing EGCG-induced changes in the WPI microenvironment. In addition, the molecular docking results indicated that hydrogen bonding and hydrophobic interactions can be generated during the binding process. Compared to the WPI and WPI–EGCG samples, the UWPI–EGCG samples exhibited higher polyphenol-binding equivalents, smaller particle sizes, and greater absolute zeta potential values. These results demonstrate that ultrasound-assisted alkali treatment enhances covalent-bonding efficiency, thereby synthesizing more stable WPI–EGCG covalent complexes than conventional alkali treatment alone. The development of walnut protein byproducts aims to achieve the high-value utilization of agricultural waste while enhancing functional properties for food and nutraceutical applications. Functional testing revealed that the preparation of WPI–EGCG covalent complexes by ultrasound-assisted alkali treatment holds significant promise for improving the solubility and emulsification capabilities of WPI. These findings not only provide theoretical support for the widespread application of WPI across various industries but also help achieve the strategic goal of transforming low-value protein residues into sustainable, market-driven functional ingredients by facilitating their high-value utilization.

## Figures and Tables

**Table 1:** The predicted binding energies for walnut protein isolate–EGCG and the amino acid residues involved in hydrogen bonds and hydrophobic contacts.

	Binding Energy (kcal/mol)	Residues Involved in Hydrogen Bonds	Residues Involved in Hydrophobic Contacts
Glutelin	–3.66	Glu 2, Ser 6, Asp 4, Val 263, Asp 264	Leu 5, Val 268, Leu 3, Phe 285, Gly 262, and Arg 266
11S globulin	–3.26	Lys 36, Pro 30, Cys 35, Gln 424	Phe 32, Gly 33, Gln 29, Arg 31, Phe 426, Glu 34, Asn 425, Pro 423, His 387, Asn 404, and Phe 405
2S albumin	–5.03	Arg 34, Asp 28, Asn 31, Glu 27	Asp 30, Arg 35, Glu 37, Gly 38, and Ile 29

## Data Availability

The original contributions presented in this study are included in the article. Further inquiries can be directed to the corresponding author.
